# Prognostic prediction of pulmonary neuroendocrine carcinoma using a transformer-based artificial intelligence model: a two-center retrospective cohort study

**DOI:** 10.3389/fonc.2026.1948262

**Published:** 2026-10-23

**Authors:** Lei Liu, Linchuan Liang, Dawei Wang, Jiaqi Zhang, Ke Zhao, Chao Guo, Cheng Huang, Shanqing Li, Yushun Gao, Yeye Chen

**Affiliations:** 1 Department of Thoracic Surgery, Peking Union Medical College Hospital, Chinese Academy of Medical Science & Peking Union Medical College, Peking, China; 2 Department of Thoracic Surgery, National Cancer Center/National Clinical Research Center for Cancer/Cancer Hospital, Chinese Academy of Medical Sciences and Peking Union Medical College, Beijing, China; 3 Institute of Advanced Research, Infervision Medical Technology Co., Ltd, Beijing, China

**Keywords:** disease-free survival, feature selection, prognosis prediction, pulmonary neuroendocrine carcinoma, survival rate

## Abstract

**Background:**

Pulmonary neuroendocrine carcinomas (pNECs) are a rare and heterogeneous group of malignancies, ranging from low-grade carcinoids to high-grade small-cell lung carcinoma (SCLC) and large-cell neuroendocrine carcinoma (LCNEC). Prognostic tools for these tumors are scarce and have rarely been validated in external cohorts.

**Objectives:**

This study aimed to develop a Transformer-based prognostic prediction model for pNEC and validate it in an independent external cohort, to improve survival estimation and support individualized treatment decisions.

**Methods:**

This retrospective two-center cohort study included 823 patients with surgically resected pNEC (2001-2023). Models were developed in Center 1 (n=374; randomly split 8:2 into training and internal test sets) and externally validated in an independent Center 2 cohort (n=449). Three algorithms—logistic regression (LR), decision tree (DT), and the Transformer-based tabular foundation model TabPFN—were compared for estimating overall survival (OS) and disease-free survival (DFS) at 1-, 3-, and 5-year landmarks, with evaluation by receiver operating characteristic, calibration, and decision curve analyses, and SHapley Additive exPlanations (SHAP) analysis for interpretability.

**Results:**

TabPFN achieved the highest area under the curve (AUC) in most tasks in both test sets, with task-dependent exceptions: in the internal test set, logistic regression performed best for 1-year OS (AUC 0.885), while TabPFN performed best for the remaining tasks, including 3-year OS (0.880), overall DFS (0.810), and 1-year DFS (0.892); in the external test set, TabPFN remained best for 1- and 3-year OS and for overall, 1-, and 3-year DFS (0.747-0.802), whereas logistic regression performed best for 5-year OS and DFS. External calibration was less satisfactory. Disease stage, pathological subtype, and age were the key prognostic predictors, and TabPFN was more accurate for low-grade subtypes. SHAP analysis quantified the contribution of each feature and made the model interpretable.

**Conclusions:**

The Transformer-based model improves survival estimation in pNEC, with task-dependent advantages over conventional methods and confirmed transportability to an independent institutional cohort. Prospective multicenter validation and calibration refinement are needed before broader clinical use.

## 1 Introduction

Pulmonary neuroendocrine carcinoma (pNEC) is a rare and heterogeneous group of tumors that includes both high-grade and low-grade neuroendocrine subtypes. These tumors differ markedly in clinical characteristics, biological behavior, and prognosis, which makes diagnosis and treatment difficult (1). Pathologically, pNECs include subtypes such as small-cell lung carcinoma (SCLC) and large-cell neuroendocrine carcinoma (LCNEC). Because the subtypes differ in molecular mechanisms and clinical behavior, management and prognostic evaluation should be subtype-specific (2).

Because of its rarity and pronounced heterogeneity, effective prognostic tools for pNEC remain scarce. Current prediction models largely depend on conventional clinicopathological parameters such as tumor size, differentiation grade, and nodal status, which may not adequately reflect the underlying biological behavior. Although deep learning models have been applied in oncology, their use in pNEC remains limited (3), and the few available prognostic models have rarely been validated in independent external cohorts, which restricts their clinical adoption. New prognostic tools that integrate multidimensional data and are validated in external cohorts are therefore needed.

Transformer models were first developed for natural language and image processing, where the self-attention mechanism proved effective at capturing complex dependencies in high-dimensional data (4). In oncology, Transformer-based approaches have shown promise, particularly for integrating clinical, genomic, and imaging data (5). However, most of these applications target high-dimensional, multimodal inputs; whether Transformer-based modeling helps in the far more common scenario of small, structured clinicopathological datasets, especially for rare tumors such as pNEC, remains unclear. The Tabular Prior-data Fitted Network (TabPFN), a pre-trained Transformer foundation model designed specifically for small-to-medium tabular datasets, performs in-context learning without task-specific hyperparameter tuning and has been reported to match or outperform gradient-boosted ensembles on small datasets; it is therefore a reasonable candidate for prognostic modeling in rare cancers.

This study aimed to develop a Transformer-based prognostic prediction model for pNEC using data from our institution and to externally validate its performance in an independent surgical cohort from a second institution. By comparing the Transformer model with conventional logistic regression and decision tree algorithms, we sought to provide a more accurate and generalizable prognostic tool to support clinical decision-making. To our knowledge, this is the first study to apply a Transformer-based tabular foundation model to prognostic prediction in pNEC and to externally validate such a model in an independent cohort.

## 2 Materials and methods

### 2.1 Patient enrollment

This retrospective study analyzed data from patients who underwent surgical treatment for pathologically diagnosed pNEC at two centers between July 2001 and May 2023. Clinical features were collected for analyses and modeling, including age, sex, smoking history, surgical methods, pathological subtypes, lymph node dissection, postoperative adjuvant treatment, survival status, tumor metastasis status, and other relevant factors. All patients were re-staged according to the 8th edition of the UICC-TNM classification system. This study was performed with authorization from the Institutional Review Board of Peking Union Medical College Hospital (S-K2117). The reporting of this study follows the STROCSS criteria (6).

After surgery, patients underwent routine surveillance including physical examinations, blood tests, and chest CT scans every 3–6 months for 5 years. Additional testing such as bone scans, head enhanced MRI, and/or PET/CT scans were conducted annually to monitor for distant metastases. Patients underwent full systemic workups if any concerning symptoms or signs appeared. Overall survival (OS) was defined as the time from surgical resection to death or time of last follow-up. Disease-free survival (DFS) was the time from surgery until locoregional recurrence, distant recurrence, or death from any cause. Based on the long-term follow-up data, eight sets of labels were created, including OS, 1-year OS (OS1), 3-year OS (OS3), 5-year OS (OS5), DFS, 1-year DFS (DFS1), 3-year DFS (DFS3), and 5-year DFS (DFS5).

Patients censored prior to each landmark time-point were excluded from the corresponding landmark analysis. For binary outcome labels, a value of 1 indicated death and 0 indicated survival for overall survival (OS) prediction. For disease-free survival (DFS), a label of 1 represented the occurrence of locoregional recurrence, distant metastasis, or all-cause death, whereas 0 indicated patients remained disease-free. All models were trained to predict the positive (label-1) event. The exact numbers of eligible patients for each task were summarized in **Supplementary Table 1**. Patients with missing survival or progression status were excluded prior to analysis. Missing values in continuous and discrete predictors were imputed using the feature-wise median calculated strictly from the training cohort, and the derived medians were then applied to the internal and external test sets to prevent cross-cohort information leakage.

### 2.2 Feature selection

Patients from Center 1 were first partitioned into training and test sets at a ratio of 8:2, and all patients from Center 2 were included in the external test set. Feature selection was performed on the training set in a multi-step manner to identify the features most relevant to OS or DFS for machine learning modeling. First, a significance test was performed on each feature in each task, and features with P < 0.05 were retained. Subsequently, all retained features were paired; if the Pearson correlation coefficient between two features exceeded 0.85, the feature with the higher P-value was removed. Finally, the least absolute shrinkage and selection operator (LASSO) method was applied to select features with non-zero coefficients from the remaining features.

### 2.3 Development of prediction models for survival status

Based on the selected features and the gold standard in each task, prediction models were first developed with conventional machine learning algorithms, including logistic regression (LR) and decision tree (DT). Because all candidate features were structured tabular variables, we used the pretrained Transformer-based tabular foundation model TabPFN (Tabular Prior-data Fitted Network) for modeling (7). TabPFN was designed for small-to-medium tabular datasets and has shown superior performance over gradient boosting methods when training samples are limited. Its in-context learning capability allows effective generalization without extensive hyperparameter tuning, which suits our relatively small pNEC cohort.

In total, eight tasks were modeled with three algorithms, and the optimal approach was selected for further analyses, including clinical usefulness, interpretability, and subgroup performance. TabPFN was deployed with N_ensemble_configurations = 10, aggregating predictions across 10 forward-pass ensemble perturbations for inference stability; LR was configured with an L2 penalty (max_iter = 1000) and DT with the standard CART algorithm (random_state = 42) as reproducible baseline configurations without additional hyperparameter tuning. For all approaches, the input was the vector of the selected clinicopathological features, and the output was the predicted probability of the positive (label-1) event for the corresponding task (death for OS tasks; locoregional recurrence, distant metastasis, or all-cause death for DFS tasks).

### 2.4 Performance evaluation and statistical analyses

Continuous variables are presented as means ± standard deviation; categorical variables as frequencies. P < 0.05 was considered statistically significant. All statistical analyses were performed with the R statistical package (The R Foundation for Statistical Computing, Vienna, Austria).

Model discrimination and calibration were evaluated with receiver operating characteristic (ROC) curves and calibration curves with the Hosmer-Lemeshow test. Clinical usefulness was assessed by decision curve analysis (DCA). The DeLong test was used to compare ROC curves between models, and the integrated discrimination improvement (IDI) index was calculated for the same purpose. Confusion matrices and Kappa coefficients were used to evaluate agreement between predictions and observed outcomes. All performance indicators were evaluated in both the training and test sets. Sensitivity, specificity, precision, negative predictive value, and F1-score were calculated at the optimal threshold determined by the Youden index, and model calibration was additionally quantified using Brier scores. SHAP (SHapley Additive exPlanations) analysis was performed to quantify the contribution of each feature to model predictions. No artificial resampling was applied; potential class imbalance was addressed at the decision-threshold level through Youden index optimization. Bootstrap resampling (1,000 iterations) was used to derive 95% confidence intervals for AUC, accuracy, sensitivity, specificity, PPV, NPV, and F1-score, and P values for pairwise model comparisons were adjusted for multiple comparisons.

## 3 Results

### 3.1 Patient characteristics

A total of 823 patients with pNEC underwent surgical treatment, with a mean age of 57.5 years (range, 14–83 years). Histologically, SCLC and LCNEC together comprised more than 50% of the cohort, while low-grade neuroendocrine carcinomas (LGNC; TC and ATC), mixed tumors, and synchronous tumors accounted for the remainder. Perioperative systemic therapy was frequently administered: 8.9% of patients received neoadjuvant chemotherapy and 62.9% received adjuvant chemotherapy and/or radiotherapy. Intraoperative frozen section analysis yielded a malignant diagnosis in 59.6% of cases, although precise classification as SCLC or neuroendocrine carcinoma was achieved in fewer than one-third. The median follow-up was 57 months (range, 1–267 months). The median OS was 57.5 months and the median DFS was 48.2 months. At the last follow-up, 453 patients were alive, 370 had died, and 287 (34.9%) had developed tumor recurrence. In total, 249 patients (30.3%) were elderly (≥65 years), and operative time exceeded 3 hours in 170 cases (20.7%). The clinical characteristics of the two cohorts are summarized and compared in **Table 1**.

**Table 1:** Baseline characteristics of enrolled patients with pulmonary neuroendocrine carcinoma in the two centers.

Variable	Center 1 (n=374)	Center 2 (n=449)	P value
Age	56.86 ± 12.78	58.12 ± 11.49	0.223
Gender			0.376
Female	107 (28.6%)	115 (25.6%)	
Male	267 (71.4%)	334 (74.4%)	
Neoadjuvant chemotherapy			<0.001
No	322 (86.1%)	428 (95.3%)	
Yes	52 (13.9%)	21 (4.7%)	
Adjuvant chemotherapy			<0.001
No	133 (35.6%)	299 (66.6%)	
Yes	241 (64.4%)	150 (33.4%)	
Adjuvant radiotherapy			0.037
No	305 (81.6%)	391 (87.1%)	
Yes	69 (18.4%)	58 (12.9%)	
Tumor Location			<0.001
Central	157 (42.0%)	173 (38.5%)	
Peripheral	210 (56.1%)	183 (40.8%)	
Indistinguishable	7 (1.9%)	93 (20.7%)	
Smoking History			0.095
No	152 (40.6%)	156 (34.7%)	
Yes	222 (59.4%)	293 (65.3%)	
Surgical approach			<0.001
Thoracotomy	120 (32.1%)	235 (52.3%)	
VATS	234 (62.6%)	202 (45.0%)	
VATS converted to Thoracotomy	20 (5.3%)	12 (2.7%)	
Resection extent			0.846
Lobectomy	262 (70.1%)	309 (68.8%)	
Combined lobectomy	78 (20.9%)	101 (22.5%)	
Sublobectomy	34 (9.1%)	39 (8.7%)	
Pathological subtype			<0.001
TC	73 (19.5%)	121 (26.9%)	
ATC	34 (9.1%)	49 (10.9%)	
SCLC	150 (40.1%)	13 (2.9%)	
LCNEC	79 (21.1%)	176 (39.2%)	
Mixed	31 (8.3%)	89 (19.8%)	
Synchronous	7 (1.9%)	1 (0.2%)	
T			0.014
T1	118 (31.6%)	115 (25.6%)	
T2	194 (51.9%)	220 (49.0%)	
T3	44 (11.8%)	86 (19.2%)	
T4	18 (4.8%)	28 (6.2%)	
N			<0.001
N0	204 (54.5%)	342 (76.2%)	
N1	72 (19.3%)	57 (12.7%)	
N2	98 (26.2%)	50 (11.1%)	
M			<0.001
0	354 (94.7%)	448 (99.8%)	
1	17 (4.5%)	1 (0.2%)	
2	3 (0.8%)	0 (0.0%)	
TNM			<0.001
I	175 (46.8%)	233 (51.9%)	
II	65 (17.4%)	125 (27.8%)	
III	118 (31.6%)	90 (20.0%)	
IV	16 (4.3%)	1 (0.2%)	

TC, typical carcinoid; ATC, atypical carcinoid; SCLC, small cell lung cancer; LCNEC, large cell neuroendocrine carcinoma.

### 3.2 Feature selection for OS and DFS prediction modeling

A multi-step approach (MSA) was used to select features for machine learning-based modeling. Particularly, age, pathological type, T category, N stage, and disease stage were all included. The LASSO coefficient-path plots are shown in **Supplementary Figure 1**.

### 3.3 Performance evaluation for OS prediction models

Model performance was better for the year-specific tasks (OS1, OS3, and OS5) than for overall OS prediction. Although the area under the curve (AUC) values declined in the external test set relative to the internal test set, they remained acceptable (**Figures 1A, C**; **Table 2**). Among the three algorithms, TabPFN achieved the highest AUC in most year-specific tasks (2/3) on both test sets (**Figures 1A, C**). Confusion matrices were also generated for each task, and the Kappa coefficients indicated fair to moderate agreement between predicted and observed outcomes for all year-specific models on the internal test set (**Figure 1B**) and for OS3 and OS5 on the external test set (**Figure 1D**).

**Figure 1:**
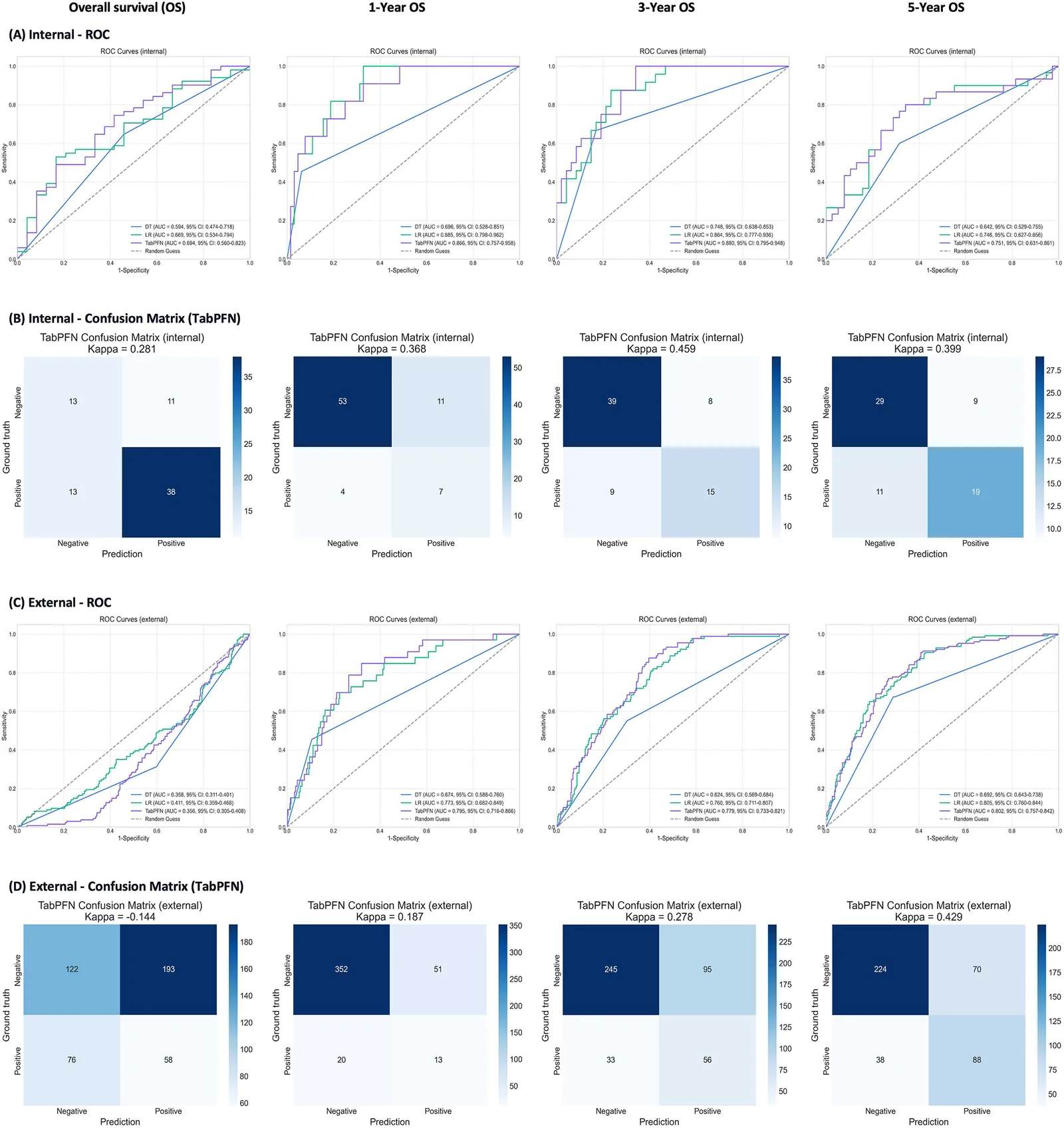
Performance evaluation of OS prediction models. **(A)** ROC curves of the three models for overall, 1-, 3-, and 5-year OS on the internal test set; **(B)** confusion matrices of the TabPFN model on the internal test set; **(C)** ROC curves on the external test set; **(D)** confusion matrices of TabPFN on the external test set.

**Table 2:** Model performance for OS and DFS prediction in the internal and external test sets.

Dataset	Task	Model	AUC (95% CI)	Accuracy (95% CI)	Sensitivity (95% CI)	Specificity (95% CI)	PPV (95% CI)	NPV (95% CI)	F1-score (95% CI)
Internal	OS	DT	0.594 (0.474 - 0.716)	61.3% (50.7% - 72.0%)	64.7% (51.8% - 76.9%)	54.2% (34.6% - 73.9%)	75.0% (62.5% - 86.7%)	41.9% (24.1% - 58.8%)	0.695 (0.581 - 0.787)
		LR	0.669 (0.534 - 0.794)	61.3% (52.0% - 72.0%)	64.7% (52.8% - 77.8%)	54.2% (33.3% - 73.9%)	75.0% (62.5% - 87.2%)	41.9% (25.0% - 59.3%)	0.695 (0.586 - 0.792)
		TabPFN	0.694 (0.560 - 0.822)	68.0% (58.6% - 78.7%)	74.5% (63.0% - 87.0%)	54.2% (33.3% - 73.7%)	77.6% (66.1% - 88.1%)	50.0% (30.4% - 69.6%)	0.760 (0.667 - 0.849)
	OS1	DT	0.696 (0.528 - 0.851) ^a^	86.7% (78.7% - 93.3%)	45.5% (12.5% - 75.0%)	93.8% (87.5% - 98.5%)	55.6% (20.0% - 90.0%)	90.9% (82.9% - 97.0%)	0.500 (0.154 - 0.737)
		LR	0.885 (0.797 - 0.961) ^b^	78.7% (69.3% - 86.7%)	81.8% (58.3% - 100.0%)	78.1% (67.2% - 87.3%)	39.1% (21.1% - 59.1%)	96.2% (90.2% - 100.0%)	0.529 (0.320 - 0.710)
		TabPFN	0.866 (0.757 - 0.958) ^b^	80.0% (70.7% - 89.3%)	63.6% (33.3% - 90.0%)	82.8% (73.4% - 90.6%)	38.9% (17.4% - 61.9%)	93.0% (84.9% - 98.3%)	0.483 (0.240 - 0.706)
	OS3	DT	0.748 (0.638 - 0.853) ^a^	77.5% (67.6% - 87.3%)	66.7% (47.4% - 85.0%)	83.0% (71.4% - 93.0%)	66.7% (45.8% - 84.6%)	83.0% (71.4% - 93.5%)	0.667 (0.488 - 0.800)
		LR	0.864 (0.777 - 0.936) ^ab^	77.5% (67.6% - 87.3%)	75.0% (57.7% - 92.0%)	78.7% (66.7% - 89.8%)	64.3% (45.5% - 81.8%)	86.0% (75.5% - 95.3%)	0.692 (0.542 - 0.828)
		TabPFN	0.880 (0.795 - 0.948) ^b^	76.1% (66.2% - 85.9%)	62.5% (44.4% - 81.0%)	83.0% (72.1% - 93.2%)	65.2% (46.1% - 84.2%)	81.2% (70.2% - 91.5%)	0.638 (0.471 - 0.778)
	OS5	DT	0.642 (0.529 - 0.755)	64.7% (52.9% - 76.5%)	60.0% (43.5% - 76.9%)	68.4% (52.8% - 82.9%)	60.0% (42.4% - 76.9%)	68.4% (52.8% - 82.9%)	0.600 (0.448 - 0.735)
		LR	0.746 (0.627 - 0.856)	70.6% (60.3% - 80.9%)	80.0% (65.5% - 93.5%)	63.2% (46.2% - 78.4%)	63.2% (48.1% - 77.8%)	80.0% (64.5% - 93.5%)	0.706 (0.571 - 0.824)
		TabPFN	0.751 (0.631 - 0.860)	70.6% (58.8% - 80.9%)	63.3% (47.0% - 80.0%)	76.3% (61.8% - 88.5%)	67.9% (50.0% - 83.9%)	72.5% (58.3% - 86.1%)	0.655 (0.510 - 0.786)
	PFS	DT	0.662 (0.557 - 0.768) ^a^	69.3% (58.7% - 78.7%)	48.4% (31.0% - 65.7%)	84.1% (72.7% - 95.2%)	68.2% (47.1% - 88.9%)	69.8% (56.9% - 81.5%)	0.566 (0.392 - 0.717)
		LR	0.803 (0.703 - 0.892) ^b^	76.0% (66.7% - 85.3%)	80.6% (66.6% - 93.8%)	72.7% (60.0% - 84.8%)	67.6% (52.8% - 81.8%)	84.2% (72.2% - 95.0%)	0.735 (0.613 - 0.842)
		TabPFN	0.810 (0.711 - 0.897) ^b^	73.3% (64.0% - 82.7%)	77.4% (61.5% - 92.3%)	70.5% (57.4% - 83.0%)	64.9% (50.0% - 78.8%)	81.6% (69.0% - 93.8%)	0.706 (0.581 - 0.819)
	PFS1	DT	0.643 (0.515 - 0.775) ^a^	74.7% (64.0% - 84.0%)	44.4% (20.0% - 68.8%)	84.2% (74.1% - 93.9%)	47.1% (25.0% - 73.3%)	82.8% (72.2% - 91.9%)	0.457 (0.231 - 0.651)
		LR	0.868 (0.789 - 0.934) ^b^	76.0% (66.7% - 85.3%)	77.8% (57.1% - 94.7%)	75.4% (63.6% - 85.2%)	50.0% (32.1% - 67.6%)	91.5% (82.0% - 98.0%)	0.609 (0.431 - 0.755)
		TabPFN	0.892 (0.810 - 0.952) ^b^	78.7% (69.3% - 86.7%)	50.0% (27.8% - 73.3%)	87.7% (78.9% - 95.0%)	56.2% (33.3% - 80.0%)	84.7% (74.6% - 93.2%)	0.529 (0.320 - 0.710)
	PFS3	DT	0.790 (0.689 - 0.885)	77.5% (67.6% - 87.3%)	80.8% (66.7% - 95.8%)	75.6% (62.2% - 87.5%)	65.6% (50.0% - 81.2%)	87.2% (76.1% - 97.0%)	0.724 (0.596 - 0.849)
		LR	0.857 (0.768 - 0.930)	74.6% (64.8% - 84.5%)	80.8% (65.0% - 95.2%)	71.1% (57.5% - 83.7%)	61.8% (45.5% - 77.4%)	86.5% (75.0% - 96.7%)	0.700 (0.553 - 0.825)
		TabPFN	0.862 (0.774 - 0.933)	77.5% (67.6% - 85.9%)	84.6% (69.7% - 96.4%)	73.3% (60.0% - 85.1%)	64.7% (48.5% - 79.4%)	89.2% (78.9% - 97.4%)	0.733 (0.596 - 0.848)
	PFS5	DT	0.758 (0.643 - 0.859)	75.0% (64.1% - 84.4%)	82.1% (66.7% - 96.2%)	69.4% (54.8% - 84.2%)	67.6% (51.6% - 82.9%)	83.3% (68.2% - 96.6%)	0.742 (0.600 - 0.852)
		LR	0.841 (0.745 - 0.926)	75.0% (64.1% - 85.9%)	78.6% (60.9% - 92.9%)	72.2% (57.7% - 86.5%)	68.8% (51.9% - 84.4%)	81.2% (68.2% - 93.3%)	0.733 (0.591 - 0.853)
		TabPFN	0.854 (0.759 - 0.936)	76.6% (65.6% - 87.5%)	71.4% (53.8% - 88.0%)	80.6% (68.6% - 92.3%)	74.1% (57.1% - 90.0%)	78.4% (64.9% - 91.2%)	0.727 (0.591 - 0.852)
External	OS	DT	0.358 (0.311 - 0.400) ^ab^	37.4% (33.2% - 41.9%)	31.3% (23.6% - 39.3%)	40.0% (34.7% - 45.5%)	18.2% (13.3% - 23.2%)	57.8% (51.1% - 64.1%)	0.230 (0.169 - 0.287)
	OS	LR	0.411 (0.359 - 0.468) ^a^	42.8% (38.1% - 47.2%)	48.5% (40.3% - 57.0%)	40.3% (34.6% - 45.1%)	25.7% (20.5% - 31.1%)	64.8% (58.0% - 71.5%)	0.336 (0.277 - 0.396)
	OS	TabPFN	0.356 (0.305 - 0.408) ^b^	40.1% (35.6% - 44.5%)	43.3% (35.0% - 51.8%)	38.7% (33.4% - 43.9%)	23.1% (18.0% - 28.2%)	61.6% (54.3% - 68.2%)	0.301 (0.241 - 0.355)
	OS1	DT	0.674 (0.588 - 0.760) ^a^	86.0% (82.3% - 89.2%) a	45.5% (29.0% - 62.5%)	89.3% (86.1% - 92.2%)	25.9% (15.3% - 37.9%)	95.2% (93.0% - 97.2%)	0.330 (0.209 - 0.453)
	OS1	LR	0.773 (0.681 - 0.848) ^ab^	75.9% (72.0% - 79.8%) b	69.7% (52.8% - 87.0%)	76.4% (72.2% - 80.7%)	19.5% (12.9% - 26.8%)	96.9% (95.0% - 98.7%)	0.305 (0.211 - 0.395)
	OS1	TabPFN	0.795 (0.716 - 0.865) ^b^	83.7% (79.8% - 86.9%) a	39.4% (22.9% - 56.3%)	87.3% (84.1% - 90.4%)	20.3% (11.1% - 29.7%)	94.6% (92.3% - 96.7%)	0.268 (0.152 - 0.376)
	OS3	DT	0.624 (0.569 - 0.683) ^a^	66.7% (62.0% - 71.3%)	55.1% (45.0% - 65.4%)	69.7% (64.4% - 74.9%)	32.2% (24.8% - 39.7%)	85.6% (80.8% - 89.6%)	0.407 (0.327 - 0.485)
	OS3	LR	0.760 (0.711 - 0.807) ^b^	70.4% (66.0% - 74.4%)	62.9% (52.5% - 72.6%)	72.4% (67.2% - 76.7%)	37.3% (29.9% - 45.1%)	88.2% (84.3% - 91.7%)	0.469 (0.389 - 0.542)
	OS3	TabPFN	0.779 (0.733 - 0.821) ^b^	70.2% (65.7% - 74.4%)	62.9% (52.7% - 72.9%)	72.1% (66.9% - 76.7%)	37.1% (29.8% - 44.5%)	88.1% (84.2% - 91.7%)	0.467 (0.384 - 0.539)
	OS5	DT	0.692 (0.643 - 0.737) a	69.5% (65.2% - 73.6%) a	67.5% (59.3% - 75.2%)	70.4% (65.1% - 75.1%)	49.4% (41.6% - 56.3%)	83.5% (79.0% - 87.9%)	0.570 (0.500 - 0.634)
	OS5	LR	0.805 (0.760 - 0.844) ^b^	67.9% (63.3% - 71.9%) a	87.3% (81.0% - 92.6%)	59.5% (53.9% - 64.7%)	48.0% (41.2% - 54.1%)	91.6% (87.7% - 95.3%)	0.620 (0.557 - 0.672)
	OS5	TabPFN	0.802 (0.757 - 0.842) ^b^	74.3% (70.2% - 78.1%) b	69.8% (61.4% - 77.9%)	76.2% (71.6% - 80.8%)	55.7% (48.1% - 62.7%)	85.5% (81.2% - 89.6%)	0.620 (0.551 - 0.680)
	PFS	DT	0.621 (0.574 - 0.670) ^a^	66.4% (62.1% - 70.8%)	49.7% (41.3% - 57.7%)	74.2% (69.1% - 79.5%)	47.3% (39.2% - 56.1%)	75.9% (71.1% - 80.4%)	0.485 (0.414 - 0.555)
	PFS	LR	0.764 (0.716 - 0.808) ^b^	69.9% (65.7% - 74.2%)	69.9% (62.2% - 77.5%)	69.9% (64.3% - 75.1%)	52.1% (45.4% - 59.0%)	83.3% (78.2% - 87.9%)	0.597 (0.536 - 0.657)
	PFS	TabPFN	0.766 (0.718 - 0.810) ^b^	68.2% (63.9% - 72.6%)	69.9% (62.5% - 77.1%)	67.3% (61.8% - 72.6%)	50.0% (43.1% - 56.8%)	82.7% (77.6% - 87.2%)	0.583 (0.518 - 0.643)
	PFS1	DT	0.586 (0.529 - 0.649) ^a^	79.1% (75.0% - 82.8%) a	29.2% (18.2% - 41.3%)	87.9% (84.4% - 91.2%)	29.7% (18.9% - 41.1%)	87.6% (84.2% - 90.8%)	0.295 (0.192 - 0.400)
	PFS1	LR	0.730 (0.671 - 0.791) ^b^	73.6% (69.5% - 77.8%) b	43.1% (30.5% - 55.7%)	79.0% (74.5% - 83.2%)	26.4% (18.1% - 34.8%)	88.8% (85.4% - 92.0%)	0.327 (0.230 - 0.420)
	PFS1	TabPFN	0.747 (0.689 - 0.806) ^b^	79.8% (76.1% - 83.5%) a	32.3% (21.6% - 44.1%)	88.1% (84.5% - 91.2%)	32.3% (20.3% - 44.1%)	88.1% (84.9% - 91.2%)	0.323 (0.217 - 0.436)
	PFS3	DT	0.600 (0.549 - 0.648) ^a^	67.3% (62.6% - 71.2%)	43.7% (34.9% - 52.6%)	76.3% (71.3% - 80.8%)	41.3% (32.3% - 50.0%)	78.0% (73.5% - 82.4%)	0.424 (0.338 - 0.504)
	PFS3	LR	0.776 (0.729 - 0.818) ^b^	71.7% (67.3% - 75.6%)	63.9% (54.1% - 72.9%)	74.7% (69.7% - 79.2%)	49.0% (40.9% - 56.6%)	84.4% (79.9% - 88.4%)	0.555 (0.479 - 0.620)
	PFS3	TabPFN	0.778 (0.731 - 0.819) ^b^	70.5% (65.9% - 74.7%)	58.8% (49.6% - 67.8%)	75.0% (70.1% - 79.7%)	47.3% (39.1% - 54.9%)	82.7% (78.2% - 87.1%)	0.524 (0.446 - 0.597)
	PFS5	DT	0.684 (0.635 - 0.728) ^a^	66.6% (62.3% - 70.9%) a	74.1% (65.9% - 81.1%)	63.1% (57.7% - 68.3%)	48.5% (41.6% - 55.0%)	83.8% (78.8% - 88.3%)	0.587 (0.522 - 0.644)
	PFS5	LR	0.787 (0.742 - 0.826) ^b^	73.2% (69.0% - 77.3%) b	66.7% (58.4% - 74.4%)	76.3% (71.1% - 80.9%)	57.0% (49.0% - 64.1%)	83.0% (78.3% - 87.2%)	0.614 (0.542 - 0.673)
	PFS5	TabPFN	0.778 (0.734 - 0.820) ^b^	71.3% (67.3% - 75.4%) b	65.9% (57.1% - 73.7%)	73.9% (69.3% - 78.6%)	54.3% (46.8% - 61.7%)	82.2% (77.3% - 86.6%)	0.595 (0.528 - 0.656)

Data are presented as value (95% confidence interval). The same superscript letter indicates that the values between models do not differ significantly from each other at the 0.05 level after correction for multiple comparisons. OS, overall survival; DFS (PFS), disease-free survival; AUC, area under the curve; PPV, positive predictive value; NPV, negative predictive value; LR, logistic regression; DT, decision tree.

We also assessed calibration by comparing predicted event probabilities with observed outcomes. As shown in **Supplementary Figure 2**, the internal calibration curves were acceptable for most models, whereas calibration deviated noticeably from ideal in the external cohort, possibly in relation to its larger sample size. For the TabPFN models, Brier scores were all below 0.188 on the external set (**Supplementary Figures 2A, B**).

### 3.4 Performance evaluation for DFS prediction models

The models showed stable performance across all DFS prediction tasks. TabPFN achieved the best AUCs for overall DFS, DFS1, DFS3, and DFS5 (0.810, 0.892, 0.862, and 0.854, respectively) on the internal test set (**Figure 2A**; **Table 2**). In the external test set, TabPFN achieved the best AUCs for overall DFS, DFS1, and DFS3 (0.766, 0.747, and 0.778), whereas LR performed best for DFS5 (0.787) (**Figure 2C**). All models showed fair to moderate agreement between predictions and observed outcomes, with minimum Kappa coefficients of 0.394 and 0.204 on the internal and external test sets, respectively (**Figures 2B, D**).

**Figure 2:**
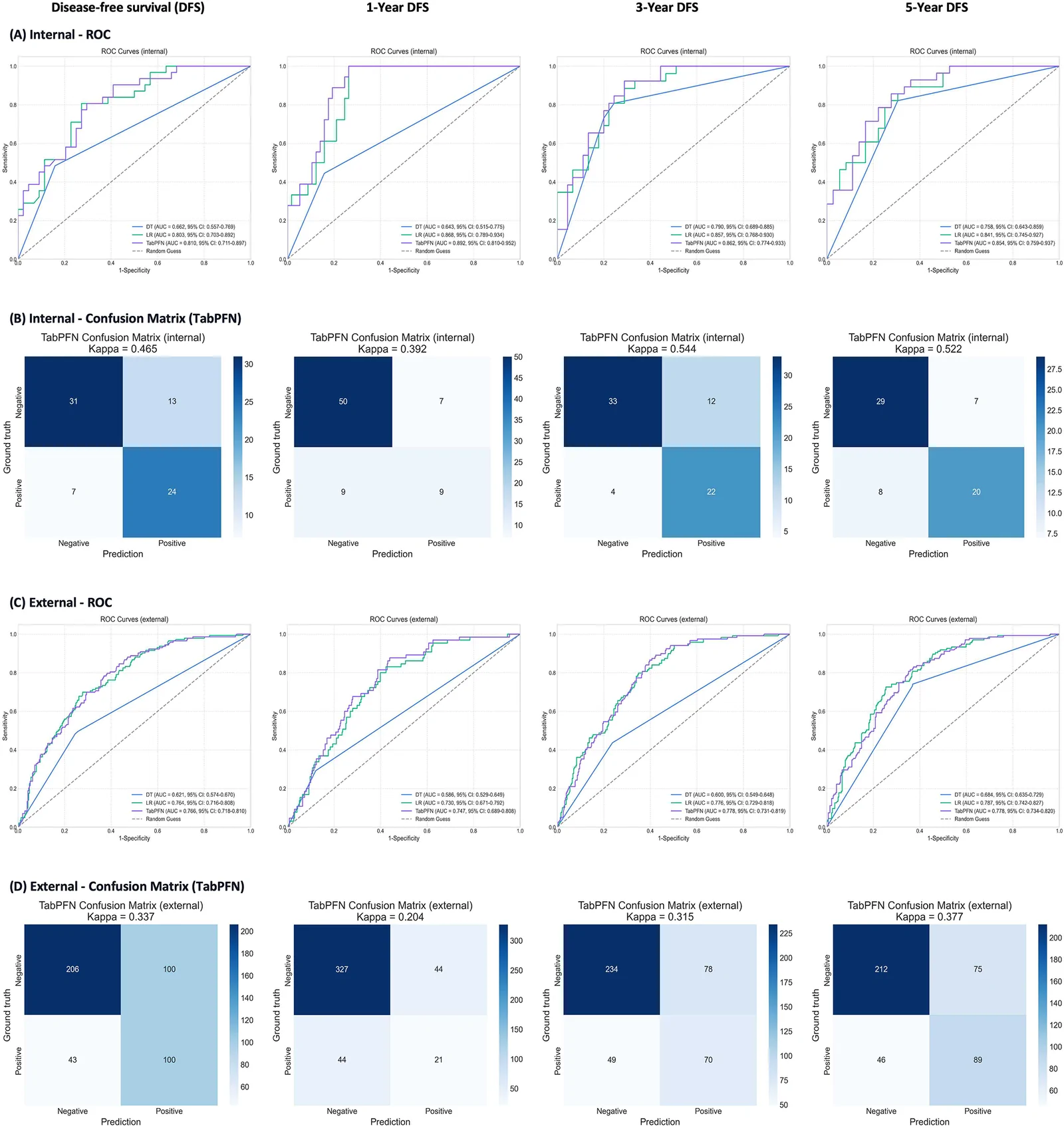
Performance evaluation of DFS prediction models. **(A)** ROC curves of the three models for overall, 1-, 3-, and 5-year DFS on the internal test set; **(B)** confusion matrices of the TabPFN model on the internal test set; **(C)** ROC curves on the external test set; **(D)** confusion matrices of TabPFN on the external test set.

Internal calibration of the DFS models was satisfactory, whereas external calibration again showed obvious deviations. For the TabPFN models, Brier scores were below 0.159 and 0.199 on the internal and external test sets, respectively (**Supplementary Figures 2C, D**).

### 3.5 Clinical usefulness assessment for the optimal OS and DFS prediction models

Based on the integrated discrimination improvement (IDI) indices across models (**Supplementary Figure 3**), TabPFN performed well in most tasks and was therefore selected as the optimal model for the DCA-based clinical usefulness assessment. TabPFN showed net benefit for all year-specific OS tasks (**Figures 3A, B**), with the largest net benefit for OS3 and OS5 on the internal and external test sets, respectively. Net benefit was generally higher for the DFS tasks (**Figures 3C, D**).

**Figure 3:**
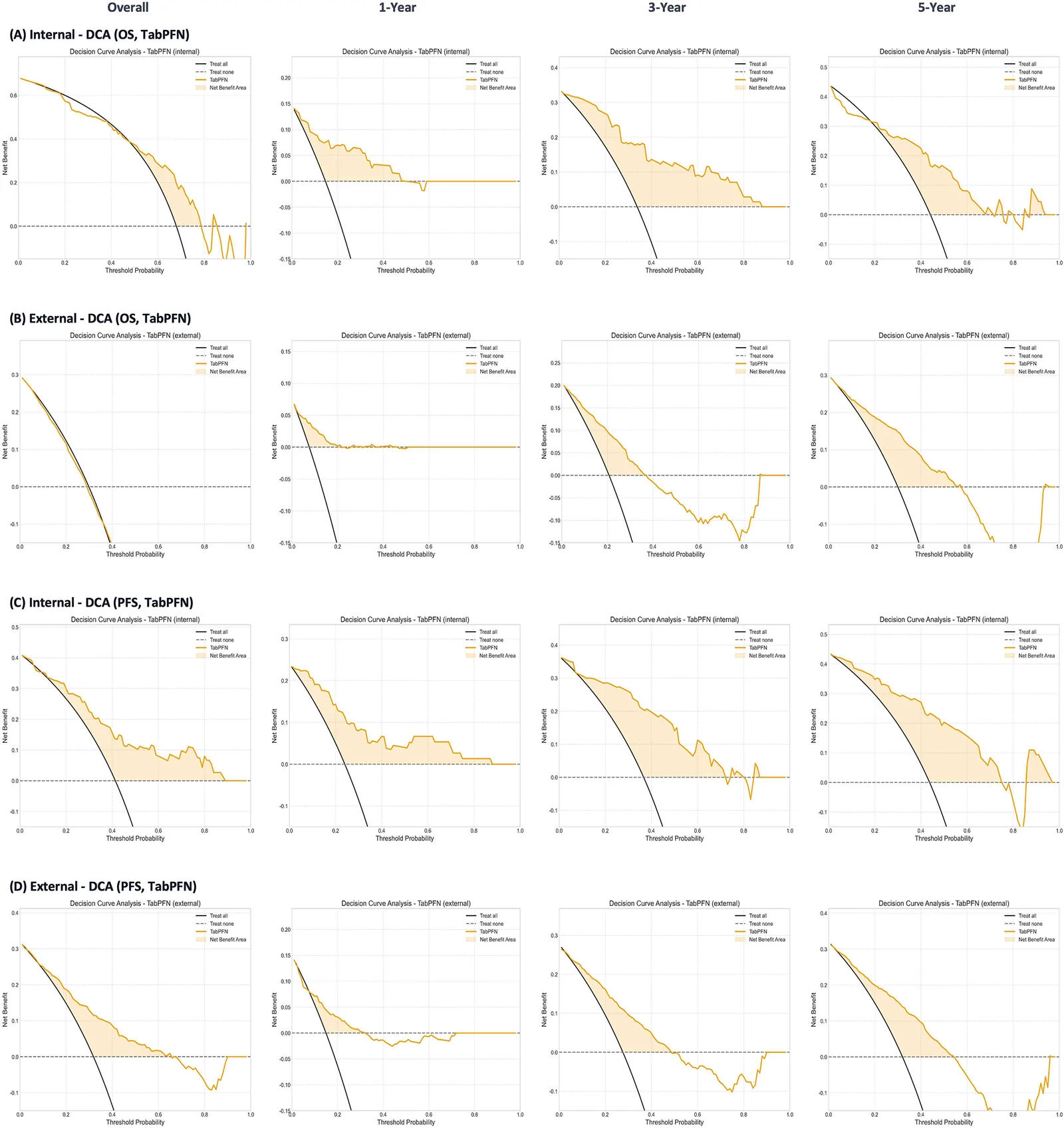
Clinical usefulness assessment of the TabPFN model via decision curve analysis (DCA). **(A)** DCA for overall, 1-, 3-, and 5-year OS on the internal test set; **(B)** DCA for OS on the external test set; **(C)** DCA for overall, 1-, 3-, and 5-year DFS on the internal test set; **(D)** DCA for DFS on the external test set.

### 3.6 Performance analyses of the optimal model on pathological subtypes

Subgroup analyses were conducted to evaluate the performance of TabPFN across pathological subtypes, including LGNC, LCNEC, SCLC, and mixed type. In the internal test set, predictive performance did not differ significantly among subtypes for OS, OS5, DFS, and DFS5; for the remaining tasks, performance was significantly better for LGNC than for the SCLC and mixed subtypes. In the external test set, TabPFN was also more accurate for LGNC in all tasks except overall OS (**Table 3**).

**Table 3:** Subgroup analysis of TabPFN accuracy across pathological subtypes in the internal and external test sets.

Dataset	Task	LGNC	SCLC	LCNEC	Mixed
Internal	OS	83.3% (66.7%–95.8%)	60.0% (43.3%–76.7%)	75.0% (50.0%–100.0%)	44.4% (11.1%–77.8%)
	OS1	100.0% (100.0%–100.0%) ^a^	70.0% (53.3%–86.7%) ^b^	91.7% (75.0%–100.0%) ^ab^	44.4% (11.1%–77.8%) ^b^
	OS3	100.0% (100.0%–100.0%) ^a^	64.3% (46.4%–82.1%) ^b^	81.8% (54.5%–100.0%) ^ab^	37.5% (12.5%–75.0%) ^b^
	OS5	82.6% (65.2%–95.7%)	57.7% (42.3%–76.9%)	90.9% (72.7%–100.0%)	50.0% (12.5%–87.5%)
	PFS	87.5% (75.0%–100.0%)	63.3% (46.7%–80.0%)	83.3% (58.3%–100.0%)	55.6% (22.2%–88.9%)
	PFS1	100.0% (100.0%–100.0%) ^a^	66.7% (50.0%–83.3%) ^b^	75.0% (50.0%–100.0%) ^ab^	66.7% (33.3%–100.0%) ^ab^
	PFS3	100.0% (100.0%–100.0%) ^a^	60.7% (42.9%–78.6%) ^b^	90.9% (72.7%–100.0%) ^ab^	50.0% (12.5%–87.5%) ^b^
	PFS5	94.7% (84.2%–100.0%)	61.5% (46.2%–80.8%)	90.9% (72.7%–100.0%)	62.5% (25.0%–87.5%)
External	OS	10.0% (5.9%–14.7%) ^a^	53.8% (23.1%–84.6%) ^b^	59.7% (52.3%–67.0%) ^b^	57.3% (47.2%–67.4%) ^b^
	OS1	99.4% (98.2%–100.0%) ^a^	84.6% (61.5%–100.0%) ^ab^	77.9% (72.1%–83.7%) ^b^	66.7% (56.3%–77.0%) ^b^
	OS3	95.7% (92.5%–98.8%) ^a^	45.5% (18.2%–72.7%) ^b^	56.5% (49.4%–64.1%) ^b^	53.5% (43.0%–62.8%) ^b^
	OS5	93.7% (89.2%–97.5%) ^a^	50.0% (20.0%–80.0%) ^b^	65.3% (58.1%–72.5%) ^b^	58.3% (47.6%–69.0%) ^b^
	PFS	85.9% (80.0%–91.2%) ^a^	53.8% (30.8%–84.6%) ^ab^	58.0% (50.6%–65.3%) ^b^	56.2% (46.1%–66.3%) ^b^
	PFS1	96.9% (93.9%–99.4%) ^a^	76.9% (53.8%–92.3%) ^ab^	69.2% (62.2%–76.2%) ^b^	69.0% (59.8%–78.2%) ^b^
	PFS3	93.8% (90.1%–97.5%) ^a^	50.0% (25.0%–75.0%) ^b^	59.4% (52.3%–66.5%) ^b^	52.9% (43.7%–63.2%) ^b^
	PFS5	90.5% (85.4%–94.9%) ^a^	63.6% (36.4%–90.9%) ^ab^	63.5% (56.3%–70.7%) ^b^	52.9% (42.4%–62.4%) ^b^

Data are presented as accuracy (95% confidence interval). The same superscript letter indicates that the accuracy of pathological subtypes does not differ significantly from each other at the 0.05 level. LGNC, low-grade neuroendocrine carcinoma (typical and atypical carcinoid).

### 3.7 Explainable analysis for the optimal OS and DFS prediction models via SHAP

SHAP analysis was performed for each task-specific model to improve interpretability. Age and disease stage ranked among the top three most important features in most prediction tasks.

For 3-year OS (OS3), for example, disease stage, pathological type, age, and T stage were the main determinants of survival, as reflected by the wide spread of their SHAP values. In the SHAP plots, red dots or bars correspond to older age or advanced disease stage and are associated with negative SHAP values, indicating a lower predicted probability of survival. This is consistent with the clinical observation that older age and advanced stage indicate poorer prognosis, and suggests that the model has learned clinically meaningful patterns. Detailed SHAP results for the other tasks are presented in **Figure 4** and **Supplementary Figure 4**.

**Figure 4:**
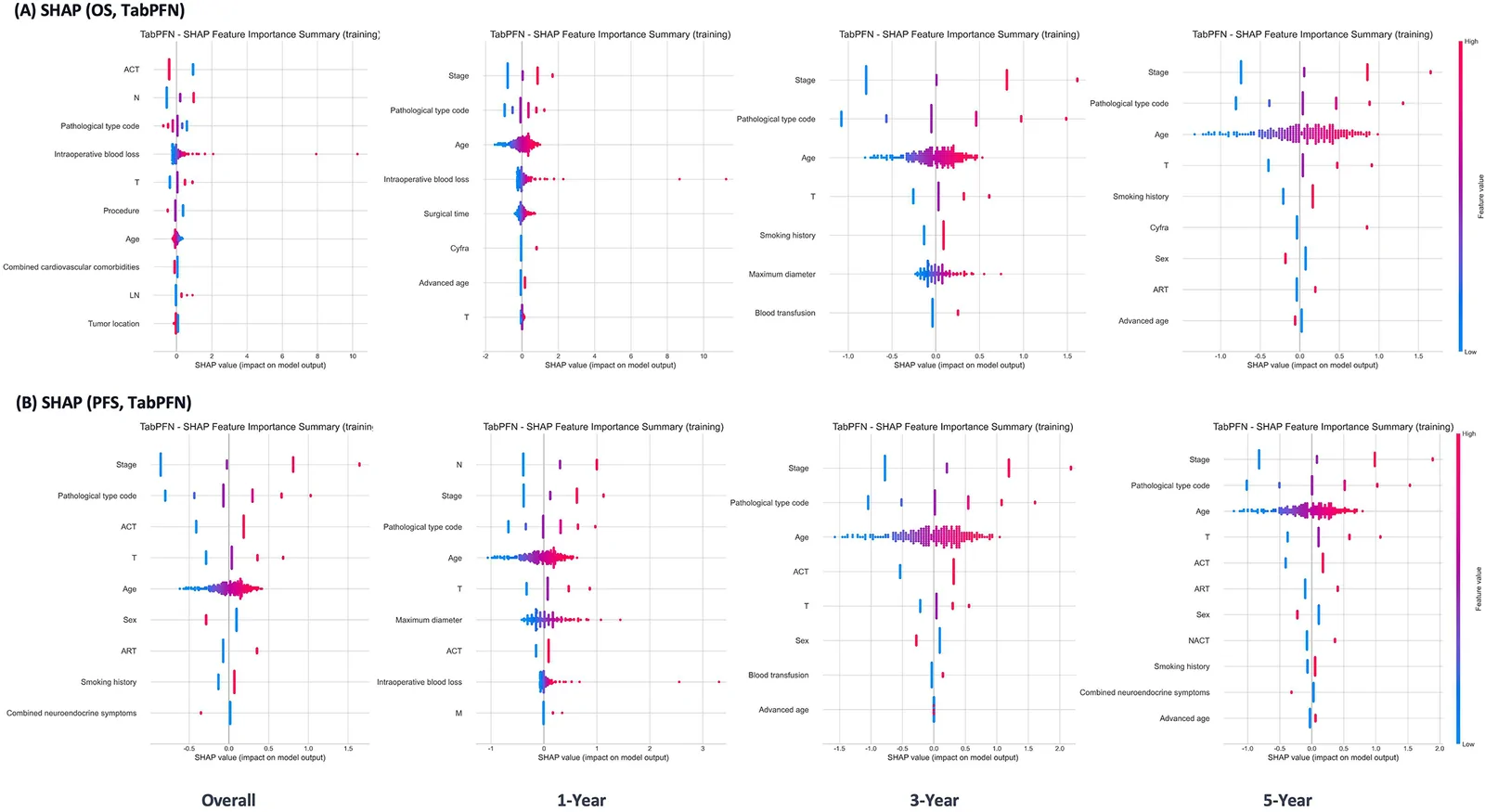
Explainable analysis of the TabPFN model via SHAP. **(A)** SHAP analysis for overall, 1-, 3-, and 5-year OS prediction; **(B)** SHAP analysis for overall, 1-, 3-, and 5-year DFS prediction.

## 4 Discussion

In this study, we developed a Transformer-based deep learning model to predict the prognosis of patients with pNEC and compared it with conventional LR and DT algorithms, using internal validation in the development cohort and external validation in an independent cohort from a second institution. The advantage of TabPFN was task-dependent rather than universal: in the internal test set, TabPFN achieved the highest AUC for overall OS, 3-year OS, 5-year OS, and all DFS tasks, whereas LR performed best for 1-year OS; in the external test set, TabPFN remained the best-performing model for 1-year and 3-year OS and for overall, 1-year, and 3-year DFS, whereas LR achieved the highest AUC for 5-year OS and 5-year DFS. SHAP analysis further improved the interpretability of the model. To our knowledge, this is the first study to develop and externally validate a Transformer-based prognostic model for pNEC. The key contributions of this study can be summarized as follows: 1) development and internal validation of TabPFN-based models for predicting OS and DFS at 1-, 3-, and 5-year landmarks in a surgical cohort of pNEC, with external validation in an independent cohort from a second institution; 2) head-to-head comparison with conventional logistic regression and decision tree algorithms using discrimination, calibration, and decision curve analyses across eight prediction tasks; and 3) task- and subtype-level performance evaluation together with SHAP-based interpretability analysis to enhance clinical transparency.

Through the self-attention mechanism, Transformer models handle high-dimensional, multimodal data well, which is advantageous for prognostic modeling in oncology (7). Transformer-based approaches have improved survival prediction in lung and gastric cancers, particularly for heterogeneous datasets derived from multiple modalities (8, 9). Pan-cancer analyses similarly indicate that Transformers offer greater scalability and predictive accuracy than logistic regression and support vector machines, especially in large patient cohorts (10, 11). Because tabular features were the primary data type in this study, we used a pre-trained Transformer architecture optimized for tabular data (TabPFN) for model development. Consistent with these reports, the Transformer model performed better than traditional machine learning models in most of our tasks. However, the computational cost and long training times of Transformers may limit their clinical adoption (12); algorithmic optimization, model compression, and parallel computing, as well as hybrid architectures combining Transformers with CNNs or LSTMs, may help improve efficiency.

TabPFN achieved the numerically highest AUC in most tasks in both the internal and external test sets, although absolute AUC values declined from the internal to the external cohort (e.g., 3-year OS: 0.880 vs. 0.779). Similar results have been reported for Transformer-based prognostic models in other cancers (13, 14). In lung cancer, Transformers have been shown to provide greater stability and predictive accuracy than conventional approaches (15). A likely reason is that self-attention captures nonlinear relationships among clinical features that conventional statistical models often miss (16). Similar advantages of deep learning models, particularly Transformers, have been reported in ovarian cancer when multidimensional data are integrated (17). Calibration, however, remains a challenge: although internal calibration was acceptable for most models, the external cohort showed obvious deviations from ideal calibration, even though the Brier scores of TabPFN remained below 0.20 across tasks. Such calibration drift is common when a model is applied in another institution with a different case mix and treatment pattern, and probability estimates may therefore need local recalibration (e.g., Platt scaling or isotonic regression) before clinical use, as reported for other deep learning models (18).

Few prognostic models have been developed specifically for pulmonary neuroendocrine carcinoma. La Salvia et al. recently proposed the Rachel score, a nomogram based on clinicopathological variables for predicting the prognosis of lung neuroendocrine tumors (19), which is currently one of the most relevant benchmarks for this disease. Compared with the Rachel score, our Transformer-based approach has complementary strengths: it captures nonlinear interactions among features through self-attention without prespecifying functional forms, requires minimal hyperparameter tuning, and provides individualized risk estimates together with SHAP-based explanations. Nomograms, in turn, are fully transparent, easy to deploy at the bedside, and less prone to overfitting in small samples. A direct quantitative benchmark against the Rachel score was not performed because several variables required by the score were not collected in our cohort; a direct comparison in external cohorts should be performed in future studies.

Three observations from the external validation deserve mention. First, although discrimination declined from the internal to the external test set, the AUC values of TabPFN for the year-specific landmark tasks remained acceptable (0.747–0.802), supporting the transportability of the model across institutions, in line with current reporting recommendations for externally validated prediction models (20). Second, the two cohorts differed significantly in case mix and perioperative management, with a predominance of LCNEC and substantially lower use of neoadjuvant and adjuvant therapy in Center 2 (**Table 1**), and the maintained performance under such distribution shift suggests that the selected clinicopathological predictors capture robust prognostic signals rather than institution-specific patterns. Third, the overall OS task, defined as death at any time during follow-up, performed poorly in the external cohort for all models (AUC < 0.5), whereas the year-specific landmark tasks transferred well. This contrast is likely explained by the dependence of the overall binary label on follow-up duration and censoring patterns, which differ between institutions, whereas landmark tasks are anchored to fixed time points and are therefore less sensitive to such differences. We therefore recommend that the landmark-based models, rather than the overall survival status model, be prioritized for clinical application.

Deep learning models are often criticized as “black boxes”, which limits their clinical acceptance. We used SHAP analysis to address this issue; it identified age, pathological subtype, T stage, and N stage as the most influential predictors of OS and DFS, consistent with studies in breast, lung, and prostate cancers (21–23). By assigning a contribution value to each feature, SHAP makes the decision process of the model transparent. For example, in the 3-year OS prediction, advanced age and higher T stage were consistently associated with poorer outcomes, in line with established clinical evidence (23, 24); such transparency may improve clinical trust and usability (25). In future work, complementary tools such as LIME and Grad-CAM could be added, particularly for multimodal prognostic tasks involving imaging data (26, 27).

The decision to pool all pathological subtypes into a single modeling framework deserves justification. First, given the rarity of pNEC, subtype-stratified modeling, particularly for mixed and synchronous tumors, would leave too few samples for stable model training. Second, pathological subtype was included as a candidate predictor in every task, allowing the models to capture subtype-dependent baseline risk while borrowing statistical strength from predictors shared across subtypes, such as stage and age. Third, the subgroup analyses (**Table 3**) provide task-level performance estimates for each subtype, allowing clinicians to gauge model reliability for an individual patient. Nevertheless, subtype-specific models, particularly separate frameworks for carcinoids versus high-grade neuroendocrine carcinomas, may achieve better performance within homogeneous populations. Developing and validating such subtype-specific models in larger multi-institutional cohorts remains an important future direction.

Predictive performance varied across pathological subtypes in the subgroup analysis, consistent with the recognized biological and clinical heterogeneity of lung neuroendocrine neoplasms (28). In both the internal and external test sets, TabPFN was generally more accurate for low-grade neuroendocrine carcinomas (LGNC; typical and atypical carcinoids) than for SCLC and mixed-type tumors. Several factors may explain this pattern. First, LGNC has a more indolent and predictable clinical course, and its outcomes are more strongly determined by stage and age, features well captured by the model, whereas outcomes of high-grade and mixed tumors depend more on molecular heterogeneity and treatment response, which clinicopathological variables alone cannot fully capture (29, 30). LCNEC, for example, exhibits substantial molecular diversity, and recent studies have identified distinct molecular subtypes (SCLC-like, NSCLC-like, and carcinoid-like) that respond differently to treatment (29). Second, mixed-type tumors by definition contain multiple histological components with potentially divergent biological behaviors, and the prognosis may be driven by the dominant component or by interactions between components, patterns that are difficult to model without component-specific quantification. Third, treatment response also differs: LCNEC is often less responsive to platinum-based chemotherapy than SCLC (30). The external subgroup estimates should be interpreted with caution given the limited subgroup sizes in the validation cohort, particularly for SCLC.

Age was also an important determinant of DFS in our study, and elderly patients had significantly poorer outcomes, consistent with previous lung cancer research (31). Advanced age is associated with diminished immune function, reduced treatment tolerance, and more comorbidities, all of which increase the risk of recurrence. Our findings agree with previous reports that age ≥70 years was significantly associated with worse DFS and OS in resected stage IB non-small cell lung cancer (32), and a population-based cohort analysis confirmed higher mortality among patients aged ≥75 years compared with those aged 65–74 years (33). We also noted that high-grade neuroendocrine carcinomas such as SCLC and LCNEC were more common in elderly patients, whereas typical carcinoid (TC) and atypical carcinoid (ATC) were more frequent in middle-aged patients (34–36); this difference in subtype distribution also contributes to the poorer prognosis of elderly patients. Beyond biological factors, treatment strategies and tolerability also affect outcomes in older patients (37). With the development of immunotherapy and targeted therapy, individualized regimens may reduce the recurrence risk in elderly patients, and age-related factors should be taken into account in prognostic models.

The model may be useful in several clinical scenarios. First, for preoperative risk stratification, it could provide preliminary prognostic estimates based on available clinical features, informing discussions about surgical candidacy and extent of resection. Second, for adjuvant therapy planning, patients identified as high-risk (e.g., predicted 3-year OS < 50%) might be considered for more aggressive adjuvant regimens or clinical trial enrollment, while low-risk patients might be spared unnecessary treatment toxicity. Third, risk-stratified surveillance protocols could be developed, with high-risk patients receiving more frequent imaging and clinical assessments. As for decision thresholds, although our DCA showed net benefit across broad threshold ranges in both test sets (largest for 3-year OS internally and 5-year OS externally), clinically meaningful thresholds should be context-specific: approximately 0.3 for identifying patients who might benefit from adjuvant chemotherapy, 0.5 for stratifying patients into intensive versus standard follow-up, and 0.7 for identifying candidates for palliative care discussions. These thresholds require prospective validation and should be refined according to institutional treatment patterns and patient preferences.

This study has several limitations. First, although external validation was performed in an independent institutional cohort, the study remains retrospective and restricted to two centers, and the two cohorts differed substantially in case mix and treatment patterns; prospective validation in broader multicenter settings is still required before clinical implementation, and subgroup generalizability is limited for subtypes underrepresented in the external cohort, particularly SCLC. Second, discrimination declined from the internal to the external test set and external calibration showed obvious deviations, indicating that transportability is not unconditional and that local recalibration (e.g., Platt scaling or isotonic regression) may be required before deployment in new settings. Third, feature selection was performed once on the training set without being embedded within resampling loops, which may introduce selection-induced optimism; the stability of the selected variables should be evaluated with resampling-based approaches in future studies. Fourth, a further limitation is the absence of genomic and radiomic features; integrating molecular markers (e.g., TP53 and RB1 alteration status, Ki-67 index, and DNA methylation profiles) and CT-based radiomic features could improve predictive accuracy, particularly for molecularly heterogeneous subtypes such as LCNEC. Fifth, although SHAP improved interpretability, additional strategies are needed to enhance transparency and clinician confidence, and integration into clinical workflows requires user-friendly interfaces and validation in hospital IT environments. Sixth, survival outcomes were modeled as binary landmark endpoints rather than by time-to-event regression (e.g., Cox proportional hazards models); this design enabled a uniform, like-for-like comparison across classification approaches, but direct comparison with Cox-based survival models is warranted in future work. Seventh, feature selection was designed primarily to satisfy the assumptions of the conventional statistical models and to keep an identical feature set across approaches for a fair comparison; Transformer-based models such as TabPFN do not require collinearity screening, and evaluating a full-feature TabPFN model may further improve performance, which we plan to explore in future work. Finally, follow-up completeness was not systematically quantified, and potential differences between patients lost to follow-up and those completing follow-up were not fully analyzed, which could introduce survival bias.

In conclusion, we developed a Transformer-based deep learning model (TabPFN) for prognostic prediction in pNEC and externally validated it in an independent institutional cohort. TabPFN achieved the best discrimination in the majority of prediction tasks, with task-dependent advantages over logistic regression and decision tree models that were most consistent for 3-year outcomes. SHAP analysis improved interpretability and provided clinicians with clinically relevant explanations to support decision-making. After external calibration refinement and prospective multicenter validation, TabPFN may become a useful tool for individualized prognostic assessment in patients with pNEC.

## Data Availability

The raw data supporting the conclusions of this article will be made available by the authors, without undue reservation.

## Supplementary material

Supplementary Figure 1 LASSO coefficient analysis in the multi-stage feature selection. **(A)** Internal, OS; **(B)** external, OS; **(C)** internal, DFS; **(D)** external, DFS.

Supplementary Figure 2 Calibration performance of the prediction models. **(A)** Internal test set, OS; **(B)** external test set, OS; **(C)** internal test set, DFS; **(D)** external test set, DFS.

Supplementary Figure 3 Comparison of integrated discrimination improvement (IDI) indices across models. The values in each cell represent the IDI between the corresponding pair of models, with color intensity reflecting the magnitude of improvement; red shades highlight significant improvements. **(A)** Internal test set, OS; **(B)** external test set, OS; **(C)** internal test set, DFS; **(D)** external test set, DFS.

Supplementary Figure 4 SHAP analysis of the TabPFN model on the internal and external test sets. **(A)** Internal, OS; **(B)** external, OS; **(C)** internal, DFS; **(D)** external, DFS.

Supplementary Table 1 Eligible patients for the OS and PFS prediction tasks.
